# Nonclinical and clinical pharmacological characterization of the potent and selective cathepsin K inhibitor MIV-711

**DOI:** 10.1186/s12967-018-1497-4

**Published:** 2018-05-09

**Authors:** Erik Lindström, Biljana Rizoska, Ian Henderson, Ylva Terelius, Markus Jerling, Charlotte Edenius, Urszula Grabowska

**Affiliations:** Medivir AB, Box 1086, 141 22 Huddinge, Sweden

**Keywords:** Cathepsin K, Osteoarthritis, CTX-I, NTX-I, CTX-II, Subchondral bone, Cartilage

## Abstract

**Background:**

Cathepsin K is an attractive therapeutic target for diseases in which bone resorption is excessive such as osteoporosis and osteoarthritis (OA). The current paper characterized the pharmacological profile of the potent and selective cathepsin K inhibitor, MIV-711, in vitro and in cynomolgus monkeys, and assessed translation to human based on a single dose clinical study in man.

**Methods:**

The potency and selectivity of MIV-711 were assessed in vitro using recombinant enzyme assays and differentiated human osteoclasts. MIV-711 was administered to healthy cynomolgus monkeys (3–30 µmol/kg, p.o.). Plasma levels of MIV-711 and the bone resorption biomarker CTX-I were measured after single dose experiments, and urine levels of CTX-I, NTX-I and CTX-II biomarkers were measured after repeat dose experiments. The safety, pharmacokinetics and pharmacodynamics (serum CTX-I) of MIV-711 were assessed in human healthy subjects after single ascending doses from 20 to 600 mg.

**Results:**

MIV-711 was a potent inhibitor of human cathepsin K (K_i_: 0.98 nmol/L) with > 1300-fold selectivity towards other human cathepsins. MIV-711 inhibited human osteoclast-mediated bone resorption with an IC_50_ value of 43 nmol/L. Single oral doses of MIV-711 to monkeys reduced plasma levels of CTX-I in a dose-dependent fashion by up to 57% at trough. The effect on CTX-I was linearly correlated to the plasma exposure of MIV-711, while the efficacy duration outlasted plasma exposure. Repeat oral dosing with MIV-711 also reduced urinary levels of the bone resorption biomarkers CTX-I (by 93%) and NTX-I (by 71%) and the cartilage degradation biomarker CTX-II (by 71%). MIV-711 was safe and well-tolerated when given as single ascending doses to healthy subjects. MIV-711 reduced serum CTX-I levels in a dose-dependent manner by up to 79% at trough. The relationship between MIV-711 exposure and effects on these biomarkers in humans was virtually identical when compared to the corresponding monkey data.

**Conclusions:**

MIV-711 is a potent and selective cathepsin K inhibitor with dose-dependent effects on biomarkers of bone and cartilage degradation in monkey and human. Taken together, MIV-711 shows promise for the treatment of bone and cartilage related disorders in humans, such as OA.

*Trial Registration* EudraCT number 2011-003024-12, registered on June 22nd 2011

## Background

Cathepsin K is a lysosomal cysteine protease predominately expressed in osteoclasts, the cells responsible for bone resorption [1]. Osteoclasts form extracellular hemivacuoles in close contact with the bone surface [2] and vesicular transport within the osteoclasts delivers cathepsin K to the hemivacuoles together with protons. While bone mineral calcium hydroxyapatite dissolves in the acidic environment, cathepsin K, which is proteolytically active at the acidic pH present in hemivacuoles [3], degrades key organic bone matrix proteins, such as type I collagen. Cleavage of type I collagen results in the release of C-terminal and N-terminal telopeptides (CTX-I and NTX-I). Cathepsin K is also expressed in chondrocytes in cartilage, and is able to cleave type II collagen and aggrecan, the main organic components of the cartilage matrix. Cartilage degradation can be assessed by measuring the C-terminal telopeptide of type II collagen (CTX-II). Although cathepsin K is expressed in other tissues, its main physiological role seems to be in bone resorption. This is reflected by the lack of bone resorption and the high bone mass phenotype in cathepsin K-deficient mice [4] and in pycnodysostosis patients, who lack functional cathepsin K due to various inactivating mutations in the cathepsin K gene [5].

The key role of cathepsin K in bone resorption makes the protease an attractive therapeutic target in disorders where bone resorption is excessive, e.g. osteoporosis and in joint diseases involving bone, such as osteoarthritis (OA). This has led to the development of several small molecule cathepsin K inhibitors with oral bioavailability and various potencies and levels of cathepsin K selectivity [6]. Cathepsin K inhibitors with sufficient potency and bioavailability reduced the biochemical biomarkers of bone resorption, NTX-I and CTX-I, which are normally released into serum/urine [7–9]. This anti-resorptive effect translated into increased bone mineral density (BMD) in ovariectomized monkeys [10, 11] and clinically in post-menopausal women [12, 13] and, in the case of odanacatib, reduced the incidence of vertebral and hip fractures in postmenopausal women in a Phase III study [14].

Cathepsin K inhibition could also be a beneficial strategy for OA since reduced subchondral bone density and quality is believed to lead to cartilage damage in OA [15, 16]. Available data in nonclinical models of joint degeneration show beneficial effects with experimental cathepsin K inhibitors on subchondral bone, cartilage and pain end-points [17–19]. Moreover, several clinical investigations have shown positive effects on cartilage integrity when subchondral bone resorption is suppressed, and a deterioration of cartilage when resorption is increased [16, 20]. Indeed, bone-acting compounds known to provide beneficial effects on bone, such as strontium ranelate [21], risedronate [22] and calcitonin [23] have also demonstrated efficacy in clinical trials on OA related endpoints, such as joint space narrowing and WOMAC scores. However, either inconsistent efficacy or safety concerns have precluded approval of these agents for clinical use in OA patients.

MIV-711 is a potent and selective cathepsin K inhibitor that is being developed as a disease-modifying treatment for patients with OA, with recent clinical data showing evidence of beneficial effects on joint structure in patients with moderate knee OA [24]. The present paper summarizes the nonclinical pharmacological profile of MIV-711 in vitro, and in cynomolgus monkeys in vivo by quantification of biomarkers of bone resorption (CTX-I and NTX-I) and cartilage degradation (CTX-II). In order to assess translation to human, the nonclinical pharmacokinetic and pharmacodynamic characteristics of MIV-711 were compared to data generated in a single ascending dose study with MIV-711 in healthy subjects.

## Methods

### Compound

The batches of MIV-711 (previously referred to as MV076159; [25]) used in nonclinical experiments were synthesized at Medivir (Huddinge, Sweden). In all nonclinical experiments in vivo, MIV-711 was suspended in 1% w/v methyl cellulose (Methocel 4AC premium, Sigma) and given as a suspension via oral gavage. The vehicle (1% w/v methyl cellulose) was administered to control animals. For the clinical study, MIV-711 was synthesized by NCK A/S (Farum, Denmark), and MIV-711 and placebo capsules were manufactured by Galenica AB (Malmö, Sweden).

### Pharmacological characterization of MIV-711 in vitro

#### Enzyme assays

Recombinant cathepsin K enzymes from all species were expressed in *E. coli*, purified and activated. Human cathepsin F and cathepsin S were expressed in *Baculovirus*, purified and activated. Purified human cathepsins B and H (Athens Research Technology), cathepsin L (Calbiochem), and cathepsin V (R & D Systems) were purchased.

H-*D*-Ala_Leu-Lys-AMC was used as the substrate in assays of cathepsin K from non-rodent species (human, dog, rabbit and guinea pig), and Z-Leu-Arg-AMC was used as the substrate in assays of cathepsin K from rodent species (mouse and rat). For cathepsins S and V the substrate used was Boc-Val-Leu-Lys-AMC, for cathepsins F and L the substrate was H-*D*-Val-Leu-Lys-AMC, for cathepsin B the substrate was Z-Arg-Arg-AMC and for cathepsin H the substrate was H-Arg-AMC. All substrates were obtained from Bachem.

For cathepsin K the buffer was 100 mmol/L sodium phosphate, 5 mmol/L EDTA, 1 mmol/L DTT, 0.1% PEG 4000, pH 6.5. For cathepsin S the buffer was 100 mmol/L sodium phosphate, 100 mmol/L NaCl, 1 mmol/L DTT, 0.1% PEG 4000, pH 6.5. For cathepsin L the buffer was 100 mmol/L sodium acetate, 1 mmol/L EDTA, 1 mmol/L DTT, 0.1% PEG 4000, pH 5.5. For cathepsin B the buffer was 50 mmol/L sodium phosphate, 1 mmol/L EDTA, pH 6.25. For cathepsin F the buffer was 100 mmol/L sodium acetate, 1 mmol/L EDTA, 1 mmol/L DTT, pH 5.5. For cathepsin H the buffer was 100 mmol/L tris–acetate, 1% PEG4000, pH 7.5. For cathepsin V the buffer was 25 mmol/L sodium acetate, 2.5 mmol/L EDTA, pH 5.5.

Assays were carried out in white polystyrene 96-well plates in a final volume of 100 µL. Substrate concentrations were 10–100 µmol/L and enzyme concentrations were 0.1–5 nmol/L. Compounds were added in DMSO in the range of 1 nmol/L–100 µmol/L at a final DMSO concentration of 1%. Plates were read in a Fluoroskan Ascent (Thermo Labsystems, Helsinki, Finland) in kinetic mode, with excitation and emission filters of 390 and 460 nm, respectively. Rates were determined by linear regression of the fluorescence/time data in Excel. Rates were fitted by non-linear regression to either the competitive inhibition equation, with the substrate concentration fixed at the value in the assay and the K_M_ fixed to the value previously determined, or the IC_50_ equation using GraphPad Prism (GraphPad, version 6, San Diego, USA) to obtain K_i_ or IC_50_ values, respectively.

The kinetics of MIV-711 inhibition of human recombinant cathepsin K were measured by progress curve analysis [26]. Briefly, 100 µmol/L substrate and 0.5 nmol/L cathepsin K were combined in 0.5 mL of buffer in a quartz cuvette and the fluorescence measured continuously in a spectrofluorometer (Hitachi F-4500, Hitachi Scientific Instruments, Woking, UK). Excitation wavelength was 385 nm and emission wavelength was 460 nm and slits were 5 and 10 nm for excitation and emission, respectively. PMT voltage was 700 V and the response time was 0.01 s. Once a linear rate was established, 5 µL MIV-711 (final assay concentration between 10 and 50 nmol/L) or 5 µL DMSO control was added and data collected until a new equilibrium rate was achieved. Data were imported into GraphPad Prism and then fitted to the Morrison equation [26] to obtain k_obs_. The k_obs_ values were plotted against the inhibitor concentration and fitted to a straight line to obtain k_on_ and k_off_.

MIV-711 was analyzed against > 70 different ion channels, receptors, transporters and cytochrome P450 (CYP) enzymes at a concentration of 10 µmol/L at PanLabs (Taipei, Taiwan).

#### Osteoclast assay

The effect of MIV-711 on osteoclast-mediated bone resorption was evaluated using a complete kit from Cambrex Bio Science (Walkersville, MD, USA). Briefly, primary osteoclasts were generated from osteoclast precursors by incubation with RANK ligand and M-CSF in 96-well plates coated with human bone fragments, according to the instructions from the provider. After differentiation (minimum of 5 days), osteoclasts were exposed to different fixed concentrations of MIV-711 for 24 h. The resorption activity of the cultures was determined by quantifying CTX-I in the culture supernatants using a commercially available enzyme-linked immunosorbent assay (CrossLaps^®^, Nordic Bioscience Diagnostics A/S, Herlev, Denmark). Controls exposed to medium without inhibitor were set to 100%. CTX-I levels from MIV-711-treated cells were normalized and expressed as % of control. Curves were generated plotting the concentration of MIV-711 versus % of control. The curves were fitted to the Hill (four parameter) equation to generate an IC_50_ value using GraphPad Prism software. The IC_50_ value was defined as the concentration of MIV-711 that reduced CTX-I levels to 50% of control.

### Nonclinical pharmacological characterization of MIV-711 in vivo

All animal studies were conducted according to the provisions of United Kingdom Law, in particular the Animals (Scientific Procedures) Act, 1986.

### Monkeys: plasma CTX-I experiments—single dose

Eight cynomolgus monkeys (Macaca fascicularis) were used for measuring plasma levels of MIV-711 and CTX-I after a single dose. The animals were healthy young males, approximately 2–4 years old and weighing 3–4 kg. Experiments were performed at Covance Ltd (Harrogate, UK). The monkeys were used over an extended period of time (up to a year) and several cathepsin K inhibitors were evaluated during this time. At least 1 week was allowed for washout in between experiments. This was not believed to affect the results with MIV-711, and vehicle controls were run on a regular basis when appropriate. Each animal received at least one dose of MIV-711 and one dose of vehicle with at least 1 week of washout in between treatments.

In single dose experiments, animals were dosed with vehicle or MIV-711 (3–30 µmol/kg) via oral gavage at a dose volume of 4 mL/kg between 7:30 and 9:00 a.m. Blood samples were collected at baseline, 30 min, 1, 2, 4, 8, 12, 24 and 48 h after dose from the femoral vein and transferred into lithium heparin anticoagulant tubes. The samples were centrifuged and the two aliquots of the resultant plasma were frozen at − 70 °C. All samples were processed and stored within 30 min of collection. The aliquots were used to determine the MIV-711 concentration and CTX-I levels, respectively.
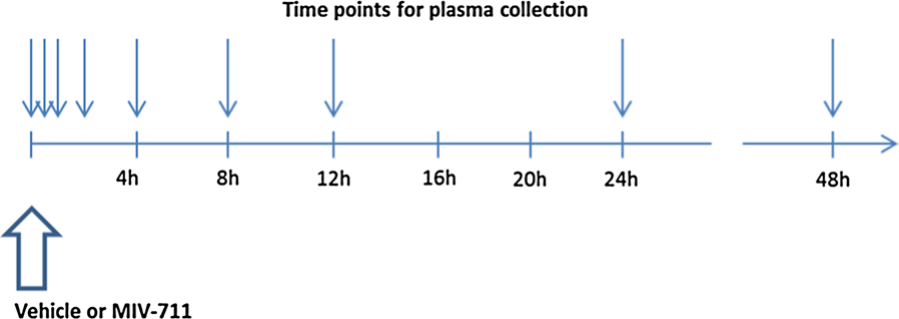


Each animal treated with MIV-711 was also treated with vehicle and sampled in an identical manner either within 2 weeks prior to MIV-711 administration, or after a washout period of between 1 and 2 weeks to enable each animal to serve as its own control in the analysis of the effects of MIV-711 on CTX-I. The area under the curve (AUC) of CTX-I during treatment with MIV-711 was divided by the AUC of CTX-I during treatment with vehicle, thus giving a % CTX-I inhibition value over 24 h. Since the concentration of MIV-711 was measured in each animal it was possible to relate the MIV-711 exposure over 24 h to the % CTX-I inhibition over 24 h in each animal.

### Monkeys: plasma CTX-I experiments—multiple doses

Repeat dosing experiments were performed in four male monkeys from the group outlined above (Covance). Vehicle or MIV-711 (30 µmol/kg) was given via oral gavage once daily for 5 days between 7:30 and 8:30 a.m. Plasma for CTX-I measurements was collected on Day 1 at baseline, 1, 2, 4, 8 and 12 h after dose, on Days 2, 3 and 4 early in the morning before next dose (i.e. at trough), and on Day 5 at trough, 1, 2, 4, 8, 12 and 24 h after dose.
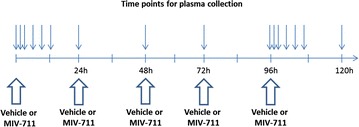


### Monkeys: urine biomarker experiments—multiple doses

Biomarkers such as NTX-I (bone resorption) and CTX-II (cartilage degradation) are typically measured in urine. Additional experiments in which urine was collected were therefore performed. In these experiments, four cynomolgus monkeys (Macaca fascicularis) were used (Aptuit, Edinburgh, UK). The animals were healthy young females weighing 3.5–4 kg. Animals were dosed with vehicle or MIV-711 via oral gavage at a dose volume of 5 mL/kg between 7:30 and 8:30 a.m. Animals first received vehicle via oral gavage for five consecutive days. After a washout period, the animals then received MIV-711 (30 µmol/kg) via oral gavage for five consecutive days.

Urine was collected between 8–12 a.m., 12 a.m.–4 p.m. and 4 p.m.–8 a.m. on Day 1 and Day 5 of respective treatment. Biomarkers were measured in each urine sample, corrected for creatinine, and the mean value of the three samples on Day 1 and Day 5 was calculated. The effect of MIV-711 was evaluated by comparing biomarkers in urine samples collected on Day 1 and Day 5 with respective vehicle sample.
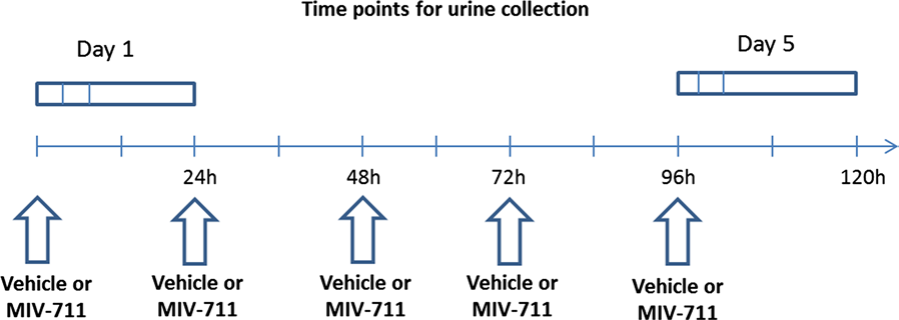


### Single ascending dose study in healthy subjects

The protocol and informed consent documentation for the clinical study was reviewed and approved by the independent ethics committee specific for the study center. The study was conducted in accordance with the International Conference on Harmonisation and Good Clinical Practice regulations and guidelines. Prior to the study, all subjects signed informed consent forms and volunteer information documents.

### Subjects

Twenty-seven healthy male and female subjects (18 male and 9 female) of any ethnic origin (24 White, 1 Asian, 1 Black, 1 Black/Caribbean White), aged between 19 and 64 years, and with a body mass index (BMI) between 18.0 and 32.0 kg/m^2^, were selected for this study. Inclusion criteria for enrollment followed the standard practice for first-in-man studies.

### Study design

This was a randomized, double-blind, placebo-controlled, sequential, single-ascending dose study conducted at a single site with the objective of evaluating the safety, tolerability, pharmacokinetics (PK) and pharmacodynamics of MIV-711. Subjects were randomized into 3 groups using a computer-generated pseudo-random permutation procedure. Each group consisted of 9 subjects with at least 3 subjects of each gender per group and subjects participated in 1–2 treatment periods. There was a minimum of 10 days between dose escalations and a minimum of 20 days between dosing occasions for individual subjects. For each subsequent dosing occasion, the decision to escalate the dose to the next level was taken after a thorough review of the safety, tolerability and PK data. In each treatment period, 7 subjects received MIV-711 and 2 received placebo (comprising of 1 subject of each gender). Dosing occurred on the morning of Day 1 following an overnight fast and subjects were kept in the fasted state until approximately 4.5 h after dosing. MIV-711 was given as a combination of 10 or 40 mg hard gelatin capsules, while placebo was given in similar capsules of identical appearance.

### Assessments

#### Safety

Standard safety assessments were performed throughout the study and included physical examination, vital signs, 12-lead electrocardiogram (ECG), continuous ECG Holter monitoring, hematology, biochemistry, urinalysis and adverse event monitoring.

#### PK

Blood samples were collected at pre-dose, 0.5, 1, 1.5, 2, 3, 4, 6, 8, 12, 16, 24, 36 and 48 h post-dose and processed into plasma for the measurement of concentrations of MIV-711. The PK analysis was conducted using WinNonlin Enterprise Version 5.2 (Pharsight Corporation, Mountain View, CA, USA).

#### Pharmacodynamic

Blood samples were collected at pre-dose, 4, 8, 24 and 48 h post-dose and processed into serum for the measurement of CTX-I concentrations.

### Bioanalysis of MIV-711

The quantification of MIV-711 in monkey plasma was performed by taking 10–50 µL of plasma and mixing with 3 volumes of acetonitrile (10 s, Vibrofix). The sample was centrifuged (10 min, 20,000×*g*, 7 °C) and 5 µL of the supernatant was injected onto the liquid chromatography with tandem mass spectrometric detection (LC–MS/MS) system. The lowest concentration of the standard curves and the lower limit of quantification (LLOQ) was 1 nmol/L of MIV-711. The human plasma samples were prepared by protein precipitation for sample extraction followed by LC–MS/MS. The lowest concentration of the standard curves and the LLOQ was 4 nmol/L of MIV-711 using 25 µL of plasma.

### Biomarker measurements

The quantification of CTX-I in monkey plasma samples and human serum samples was performed using a commercially available kit (Serum CrossLaps ELISA, IDS Nordic, Herlev, Denmark). The levels of CTX-I in monkey urine samples were measured using a commercially available kit (Urine BETA CrossLaps ELISA, IDS Nordic). The quantification of NTX-I in monkey samples was performed using a commercially available kit (Osteomark NTx Urine, Wampole Laboratories, Inc, Princeton, USA). Urinary levels of CTX-II in monkey samples were measured using a commercially available kit (Urine CrossLaps EIA, IDS Nordic). The concentrations of biomarkers in the urine samples were corrected for creatinine concentrations. The creatinine levels in urine were determined at Swedish University of Agricultural Sciences, using the enzymatic assay (cat no. 8L24-01) on the Abbott system Architect c4000.

### Statistical analysis

In the nonclinical studies, two-way ANOVA was used to assess the effects of MIV-711 on plasma CTX-I. Pearson’s correlation was used when comparing plasma exposure of MIV-711 with inhibitory effects on CTX-I. Student’s unpaired *t* test was used when assessing the effect of MIV-711 on urine biomarkers. In the clinical study, PK parameters were summarized by dose level using descriptive statistics. The dose proportionality for AUC and C_max_ was assessed by using a power model with determination of the linear regression slope with a 95% confidence interval. Change in CTX-I from baseline at 24 h after dose was calculated by dose level and summarized using descriptive statistics. In addition, the 95% confidence interval for the difference between each MIV-711 group and the placebo group for these variables was calculated by dose level. Pearson’s correlation was used when comparing plasma exposure of MIV-711 with inhibitory effects on CTX-I. A time-matched and placebo-corrected approach was used to evaluate the effects of MIV-711 on the QTcF interval from the Holter ECG recordings. The change-from-baseline QTcF at post-dose time points was analyzed using an ANCOVA model including the treatment, time (categorical), and treatment-by-time interaction as fixed effects, baseline QTcF as a covariate, and subject as random effect. A *p* value < 0.05 was considered statistically significant. Data are expressed as mean ± SEM.

## Results

### Pharmacological characterization of MIV-711 in vitro

#### Inhibition of human cathepsin K and related proteases

MIV-711 had a mean K_i_ (dissociation constant) value of 0.98 nmol/L for human cathepsin K (Table 1). Selectivity was more than 1300-fold versus the other human cathepsins tested. The catalytically active forms of human and cynomolgus monkey cathepsin K have identical amino acid sequences and there are no known post-translational modifications in the active forms [27]. Therefore, the catalytically active forms of the human and cynomolgus cathepsin K enzyme are regarded as identical. MIV-711 was also a potent inhibitor of dog, guinea pig and rabbit cathepsin K (Table 1). In contrast, MIV-711 was found to be a substantially less potent inhibitor of rat and mouse cathepsin K enzymes, with IC_50_ values of 240 and 1000 nmol/L respectively.

**Table 1:** Potency of MIV-711 against various cathepsin enzymes

Species	Recombinant enzyme	K_i_ (nmol/L)	95% CI (nmol/L)	n
Human	Cathepsin K	0.98	0.88–1.09	40
	Cathepsin B	1300		1
	Cathepsin L	1700	1500–1900	39
	Cathepsin F	2800		1
	Cathepsin V	4000		1
	Cathepsin H	> 10,000		1
	Cathepsin S	15,700	12,500–19,800	39
Dog	Cathepsin K	1.5	0.75–2.9	4
Rabbit	Cathepsin K	3.3^a^	0.9–12	4
Guinea pig	Cathepsin K	9.8	8.1–12	2
Rat	Cathepsin K	240^a^	180–320	3
Mouse	Cathepsin K	1000^a^	680–1500	3

K_i_ values are geometric means. 95% CI—geometric 95% confidence interval

^a^IC_50_

The k_on_ and k_off_ rates that characterize the binding of MIV-711 to human recombinant cathepsin K were evaluated in an isolated enzyme assay in vitro. The k_on_ rate of MIV-711 to human cathepsin K was 4.6 × 10^6^ M^−1^s^−1^ and the k_off_ rate was 8 × 10^−3^ s^−1^. The k_off_ rate translates to a residence time of about 90 s. From these experiments, the K_i_ value for MIV-711 was 1.7 nmol/L, consistent with the value of 0.98 nmol/L obtained using the equilibrium method above.

No significant responses (more than 50% inhibition) were recorded when MIV-711 was tested against a broad panel of > 70 different ion channels, receptors, transporters and CYP enzymes at a concentration of 10 µmol/L.

#### Inhibition of osteoclast-mediated bone resorption

**Fig. 1:**
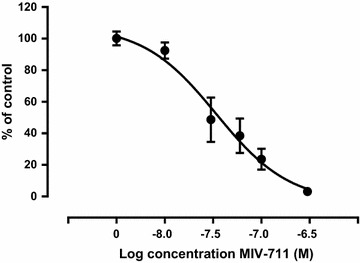
Effect of MIV-711 on human osteoclast-mediated bone resorption using human bone fragments. Control cells exposed to medium without inhibitor were set to 100%. CTX-I levels from MIV-711-treated cells were normalized and expressed as % of control. Data are from two osteoclast preparations with each preparation measured in triplicate (n = 6). The data were fitted to the Hill equation. Mean ± SEM

The inhibition of bone resorption in vitro by MIV-711 was studied using differentiated human osteoclasts incubated together with human bone fragments. MIV-711 inhibited human osteoclast activity in a concentration-dependent manner with a geometric mean IC_50_ value of 43 nmol/L (Fig. 1). The results are consistent with a previous study, in which the IC_50_ value of MIV-711 in a bone resorption assay using differentiated human osteoclasts incubated together with bovine bone slices was 37 nmol/L [25] (note: MIV-711 was referred to as MV076159 in that publication).

### Pharmacological characterization of MIV-711 in cynomolgus monkeys in vivo

#### Effect of single oral doses of MIV-711 on plasma CTX-I levels

**Fig. 2:**
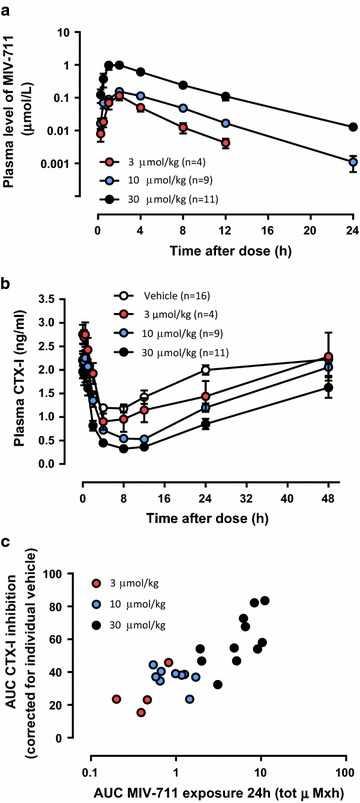
**a** MIV-711 concentrations in monkey plasma at various time points after a single dose. **b** Plasma CTX-I levels measured in the same samples as in **a**. **c** Relationship between MIV-711 exposure (AUC_0–24h_) and % inhibition of plasma CTX-I over 24 h. Mean ± SEM

Healthy, male cynomolgus monkeys were treated with vehicle or MIV-711 at doses of 3, 10 or 30 µmol/kg by oral gavage. The results, shown in Fig. 2a and Table 2, demonstrate that the administration of increasing doses of MIV-711 resulted in a dose-proportional increase in plasma levels of MIV-711. T_max_ ranged between 1 and 3 h and t_1/2_ ranged between 2 and 4 h. The C_max_ reached with the highest dose was approximately 1 µmol/L. At 24 h post-dose, MIV-711 was no longer detectable (< LLOQ of 1 nmol/L) in the plasma of animals in the lowest dose group, but it was detectable in some animals in the intermediate dose group, while the plasma concentration of MIV-711 was approximately 10 nmol/L in the highest dose group.

**Table 2:** Pharmacokinetic parameters in monkey following single oral doses of MIV-711

Dose (µmol/kg)	C_max_ (µmol/L)	AUC_0–t_ (µmol/L × h)	AUC_inf_ (µmol/L × h)	t½ (h)	t_max_^a^ (h)	C_max_/dose	n
3	0.102 (65)	0.400 (60)	0.409 (62)	2.1 (16)	2.0 (2.0–2.0)	0.034 (65)	4
10	0.164 (41)	0.881 (52)	0.932 (46)	3.1 (52)	2.0 (0.50–4.0)	0.016 (41)	9
30	0.923 (56)	6.21 (62)	6.28 (62)	3.4 (30)	2.0 (1.0–4.0)	0.031 (56)	11

Data are presented as geometric mean (CV%)

^a^Median (min–max)

Corresponding plasma CTX-I levels are shown in Fig. 2b. In the vehicle-treated animals, the normal diurnal rhythm in bone resorption was observed, with plasma CTX-I levels being 47% lower than baseline at 8 h post-dose but having returned to baseline levels at 24 h post dose (Fig. 2b). MIV-711 reduced plasma CTX-I levels in a dose-dependent manner. The highest dose of MIV-711 reduced CTX-I levels by 57% compared to baseline at 24 h post-dose. This CTX-I lowering effect was statistically significant relative to the vehicle groups at all time points between 2 and 24 h post-dose (*p* < 0.05). The effect of the mid-dose MIV-711 (10 µmol/kg, p.o.) was statistically significant versus vehicle between 8 and 24 h post-dose (*p* < 0.05). CTX-I levels after treatment with the low dose MIV-711 (3 µmol/kg, p.o.) did not differ significantly vs. vehicle (*p* > 0.05). The decreased CTX-I levels were approaching baseline levels in all dose groups at 48 h post dose, indicating that the pharmacological effect of a single dose was fully reversible.

The % CTX-I inhibition over 24 h versus the MIV-711 exposure over 24 h for each individual animal is shown in Fig. 2c. There was a highly significant correlation between plasma exposure of MIV-711 and effects on bone resorption (*p* < 0.0001, Pearson correlation, r^2^ = 0.71).

#### Effect of repeated oral doses of MIV-711 on plasma CTX-I levels

**Fig. 3:**
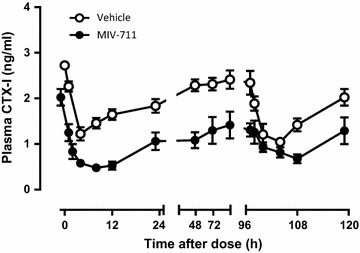
Plasma CTX-I concentrations in response to repeat oral dosing once daily for 5 days with vehicle or MIV-711 (30 µmol/kg). Mean ± SEM, n = 4

Repeat oral dosing with vehicle or MIV-711 (30 µmol/kg, p.o.) once daily in the morning for 5 days resulted in similar reductions in plasma levels of CTX-I on Day 5 as on Day 1 (Fig. 3). On Day 1, the maximum effect of MIV-711 on plasma CTX-I levels was observed at 12 h post dose (76% reduction from baseline), while on Day 5 the maximal effects were 67% lower than the initial baseline before the first dose. The trough levels of CTX-I in plasma were 38% lower than baseline on Day 5 (compared to 48% after the first dose on Day 1). The trough concentrations of CTX-I in plasma were statistically significantly reduced on each day after MIV-711 treatment compared to the vehicle control (*p* < 0.05).

#### Effect of repeated oral doses of MIV-711 on urine CTX-I, NTX-I and CTX-II levels

**Fig. 4:**
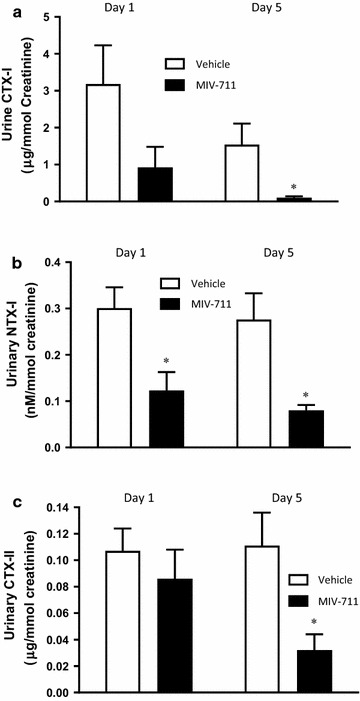
Urine biomarker concentrations in response to single or repeat oral dosing once daily for 5 days with vehicle or MIV-711 (30 µmol/kg). Biomarkers in the graph were measured in urine samples collected between 0–4, 4–8 and 8–24 h after dose on Day 1 and Day 5 of vehicle and MIV-711 treatment. The mean value of the concentrations measured in the three samples collected was calculated. Mean ± SEM, n = 4. *p < 0.05 versus corresponding vehicle

The effect of MIV-711 on creatinine-corrected levels of CTX-I, NTX-I and CTX-II in urine are shown in Fig. 4. NTX-I and CTX-II levels did not change after 5-day treatment with vehicle, whereas CTX-I levels were numerically lower on Day 5 compared to Day 1 (*p *> 0.05). A single dose of MIV-711 immediately reduced urinary CTX-I and NTX-I levels compared to vehicle while CTX-II levels were unaffected. Repeated dosing with MIV-711 for 5 days reduced urinary CTX-I, NTX-I and CTX-II levels by 93% (*p* < 0.05), 71% (*p* < 0.05) and 71% (*p* < 0.05) compared to vehicle, respectively.

### Single ascending doses of MIV-711 in healthy subjects

In this study, 27 subjects participated and received either MIV-711 or placebo on two separate occasions with a minimum interval between doses of 20 days.

### Safety

MIV-711 was safe and well-tolerated when given as single ascending doses to healthy subjects. There were no serious adverse events and no subjects discontinued the study. There were no apparent dose-dependent trends in clinical laboratory data, vital signs, and ECG parameters. Holter ECG monitoring demonstrated that there was no clinically relevant effect on the QTcF interval. After administration of the maximum dose of 600 mg, the upper confidence interval of the projected QTcF effect at the highest observed plasma levels was below 10 ms. Adverse events were reported by 5/10 (50%) placebo-treated subjects, and by 11/35 (31%) MIV-711-treated subjects. In placebo-treated subjects, 2/10 (20%) of the reported adverse events (one mild, one moderate) were considered possibly related to treatment (one headache, one rash) while in MIV-711-treated subjects, 6/35 (17%) of the reported adverse events (all mild) were considered possibly related to treatment (two hot flush, one acne, one rash, one cough, one headache).

### Pharmacokinetics

**Fig. 5:**
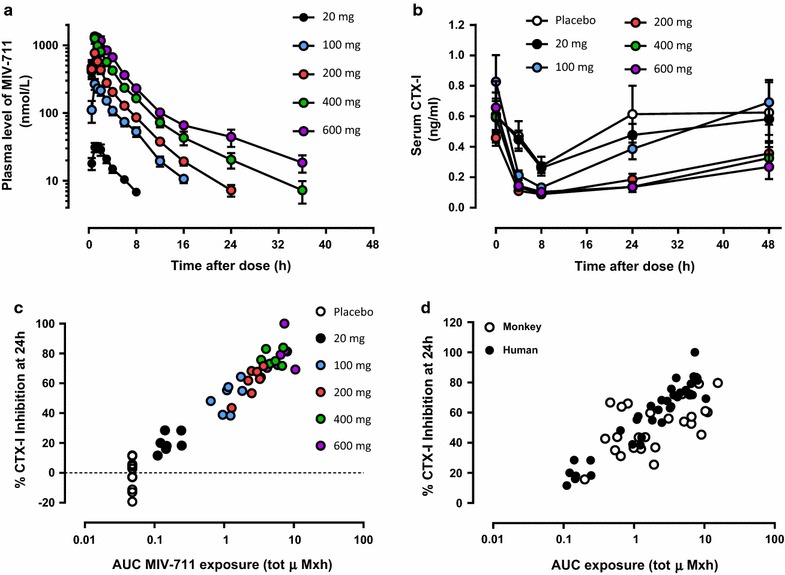
**a** MIV-711 concentrations in human plasma at various time points after a single dose. **b** CTX-I levels were measured in serum at various time points after a single dose. **c** Relationship between MIV-711 exposure (AUC_0–24h_) and % inhibition of serum CTX-I at 24 h post-dose. **d** Exposure-efficacy comparison between monkey and human. Grouped data are expressed as Mean ± SEM, n = 7–10

Figure 5a and Table 3 demonstrate that the systemic exposure of MIV-711 was dose-proportional over the single dose range of 100–600 mg, and supra-proportional between 20 and 100 mg. The slope for dose proportionality was 1.12 for AUC_0–inf_ (95% CI 1.05–1.19) and 1.14 for C_max_ (95% CI 1.01–1.26) even when including the 20 mg group. MIV-711 was rapidly absorbed with a median t_max_ of 1 h and was eliminated in a biphasic manner with a mean terminal t_1/2_ of 3.4–8.3 h over the 20–600 mg dose range. MIV-711 was extensively metabolised with less than 1% excreted renally unchanged (*data not shown*).

**Table 3:** Pharmacokinetic parameters in human following single oral doses of MIV-711

Dose (mg)	C_max_ (µmol/L)	AUC_0–t_ (µmol/L × h)	AUC_inf_ (µmol/L × h)	t½ (h)	t_max_^a^ (h)	C_max_/dose	n
20	0.033 (36)	0.128 (36)	0.158 (32)	3.4 (13)	1.0 (0.50–2.0)	0.12 (26)	7
100	0.293 (35)	1.13 (38)	1.17 (37)	3.7 (15)	1.0 (1.0–2.2)	0.21 (23)	7
200	0.756 (43)	2.45 (36)	2.51 (35)	5.0 (16)	1.0 (0.50–1.0)	0.27 (36)	7
400	1.18 (43)	4.62 (33)	4.75 (32)	6.5 (44)	1.0 (1.0–1.5)	0.23 (37)	7
600	1.39 (37)	6.75 (30)	6.89 (30)	8.3 (34)	1.0 (1.0–3.0)	0.17 (38)	7

Data are presented as geometric mean (CV%)

^a^Median (min–max)

### Pharmacodynamics

Following administration of placebo, a diurnal variation in serum CTX-I levels was evident, similar to previous studies [28], with levels steadily declining from around 8:45 a.m. until 4:45 p.m. (8 h post-dose administration), when the lowest levels were observed (Fig. 5b). CTX-I levels returned to baseline over the next 16 h. MIV-711 treatment reduced nadir CTX-I levels further, and CTX-I levels at 24 h post dose were decreased in a dose-dependent manner (Fig. 5b). The effect of MIV-711 at 24 h ranged from 20% inhibition versus baseline (20 mg dose) to 79% inhibition versus baseline (600 mg dose). When compared to placebo, the reduction in serum CTX-I levels at 24 h post-dose achieved statistical significance at all dose levels. At 48 h post-dose, CTX-I levels were returning towards baseline levels but were still somewhat reduced in subjects receiving 200–600 mg MIV-711. Figure 5c demonstrates that increased exposure of MIV-711 in humans reduces CTX-I levels in an exposure-dependent manner. The exposure-effect relationship with MIV-711 was similar when comparing monkey data with human data, the free fraction of MIV-711 being similar in human and monkey (Fig. 5d). There were highly significant correlations between plasma exposure of MIV-711 and the effects on this biomarker of bone resorption (human: *p *< 0.0001, r^2^ = 0.58; monkey: *p* = 0.0007, r^2^ = 0.39). The exposures required for 50% reduction of CTX-I at 24 h post dose were 1.3 and 1.5 µmol/L × h for human and monkey, respectively.

## Discussion

Osteoarthritis is a disease characterized by cartilage degradation and increased turnover of bone. Treatments for patients with OA are currently limited to managing the symptoms of the disease. As cathepsin K is the principal protease responsible for the degradation of collagen in both bone and cartilage, cathepsin K inhibition represents an attractive approach to modify the course of the disease. The present paper summarizes the nonclinical pharmacological profile of the cathepsin K inhibitor MIV-711 in vitro and in vivo, and translation of PK profile and pharmacodynamic effects to human in a single ascending dose study with MIV-711 in healthy subjects as the first step towards the selection of the MIV-711 doses to be used in a proof of concept study in OA patients.

MIV-711 displayed high potency against recombinant human cathepsin K enzyme (K_i_: 0.98 nmol/L) and inhibited human osteoclast-mediated bone resorption with an IC_50_ of 43 nmol/L. The observation that concentrations of MIV-711, which has a pKa value of 7.15, need to be around 50-fold higher than the K_i_ value to show effects on bone resorption in vitro is consistent with previous data [25].

In monkeys, MIV-711 reduced plasma CTX-I concentrations in a dose-dependent manner after oral administration with a highly significant exposure-effect correlation. Interestingly, MIV-711 reduced plasma CTX-I levels by over 50% at 24 h post-dose despite plasma levels of MIV-711 being very low at this time point (mostly < 10 nmol/L). This suggests a prolonged inhibitory effect of MIV-711 on bone resorption in vivo at relatively low plasma concentrations (AUC_0–24h_: 1.5 µmol × h/L for 50% reduction). The prolonged effect is most likely not due to a long residence time of MIV-711 to the cathepsin K enzyme since the off rate was found to be relatively rapid. Fuller et al. [25] previously demonstrated that non-acidic (MIV-711) and acidic (odanacatib) cathepsin K inhibitors had similar anti-resorptive potencies in an in vitro assay of human osteoclast-mediated bone resorption, and had similar rapid off-rates from the human cathepsin K enzyme. However, the inhibitory effect MIV-711 on osteoclast function lasted for a significantly longer time than odanacatib after washout, although the effects were fully reversible in both cases. This advantage for non-acidic inhibitors like MIV-711 was attributed to its positive charge in the acidic hemivacuoles, which results in reduced permeation out of the hemivacuole and thereby a longer residence time. The current paper extends these findings by demonstrating that the relatively long-lasting pharmacological effect of MIV-711 in vitro is also observed in vivo.

Repeat dosing of MIV-711 to monkeys also decreased urinary levels of CTX-I and NTX-I. This is to be expected in response to successful target engagement, since cathepsin K is known to generate these telopeptide crosslinks directly from type I collagen via enzymatic cleavage [29, 30]. MIV-711 reduced urinary NTX-I levels in monkeys by 71%, which is comparable to other cathepsin K inhibitors that have been investigated in clinical studies [9, 12, 13]. An approximate 70% reduction of urine NTX-I seemed to be the maximal effect that could be attained through cathepsin K inhibition. Presumably, urinary NTX-I can be produced from other tissues besides bone through a cathepsin K-independent manner. By contrast, urinary CTX-I levels were reduced by 93%. This profile of effects on biomarkers, with almost complete reduction of CTX-I and 60–70% reduction of NTX-I, has been shown in humans with other cathepsin K inhibitors in postmenopausal women including MIV-711 [9, 13, 31]. Doses of cathepsin K inhibitors that have provided these degrees of urinary NTX-1 and CTX-I reductions have also significantly improved bone mineral density in humans [12, 13]. Urinary levels of CTX-I were reduced much more in monkeys in response to MIV-711 treatment compared to plasma levels of CTX-I. This is similar to human data reported by others [9, 13]. The reason for this is unclear, but may reflect cathepsin K-independent CTX-I-like immunoreactivity which appears to be present in plasma but not in urine.

MIV-711 also reduced urinary levels of CTX-II by 71% following repeat dosing to monkeys. CTX-II is a C-terminal telopeptide derived from collagen type II, the main structural component of articular cartilage. Cathepsin K has the ability to cleave collagen type II in vitro and clinical data show up-regulation of cathepsin K expression in the joints of OA patients [32, 33]. However, the cathepsin K-mediated cleavage sites appear to be mainly located in the triple helix of type II collagen [34, 35] while the CTX-II epitope is released from cartilage by matrix metalloproteinases [36]. In clinical studies, the cathepsin K inhibitor ONO-5334 reduced urinary CTX-II by 50% after treatment of postmenopausal women with osteoporosis for 12 months [13]. Similarly, we have shown that 100 mg MIV-711 once daily reduces urinary CTX-II levels by 55% after treatment of postmenopausal women for 1 month [31] and by 72% in healthy subjects after 200 mg MIV-711 daily for 7 days [37]. Interestingly, anti-resorptive therapies, which are not expected to directly affect articular cartilage, such as strontium ranelate, risedronate and calcitonin, also reduce urinary levels of CTX-II in OA patients [21–23]. Hence, the effects of MIV-711 on CTX-II levels may be mediated by cathepsin K engagement directly in cartilage or indirectly by engaging cathepsin K in subchondral bone or both mechanisms could be involved. In the current paper, urinary CTX-II levels were largely unaffected by a single dose of MIV-711, while bone resorption biomarkers were immediately reduced. After treatment with MIV-711 for 5 days, CTX-II levels were also reduced in the monkeys. This apparent delay in CTX-II reduction could suggest an indirect mechanism, but could also be due to differences in the turnover of these biomarkers. Nonetheless, CTX-II is the most frequently used biomarker for assessing cartilage degradation in OA and has been useful for assessing disease burden, predicting progression and has been reduced in several clinical trials of drugs that were shown to have a beneficial effect on structural endpoints [38, 39]. The CTX-II data suggests that MIV-711 may be useful in reducing subchondral bone turnover and attenuating cartilage disease progression in OA patients. Indeed, MIV-711 has demonstrated protective effects on subchondral bone and articular cartilage in nonclinical disease models [40] at exposures that are in the same range as reported in this study. Additionally, recent clinical data from a Phase II study with MIV-711 in knee OA patients, given once daily for 6 months, demonstrated benefit on joint structure, with significantly lower increases in bone area and cartilage thinning in the diseased knee, as assessed by magnetic resonance imaging (MRI), compared to patients who received placebo [24].

The prolonged pharmacodynamic effect on plasma CTX-I, and the exposure-effect correlation observed in monkey appeared to translate well to humans where relatively low plasma concentrations of MIV-711 were sufficient for a given anti-resorptive efficacy. In humans, more than 50% reduction of serum CTX-I was attained at 24 h after single doses of 100 mg and higher; a time point when plasma concentrations of MIV-711 were close to, or below, LLOQ (< 4 nmol/L). For instance, 100 mg MIV-711 evoked a 54% reduction of CTX-I at 24 h post-dose, a time point when the plasma levels were mostly < 4 nmol/L. The AUC_0–24h_ at 100 mg MIV-711 was 1.1 µmol × h/L (Table 3) which is consistent with nonclinical experiments, in which an AUC_0–24h_ of 1.5 µmol × h/L was required for 50% CTX-I reduction at trough. In contrast to MIV-711, the trough plasma IC_50_ for odanacatib in human when given at a weekly dose of 50 mg (the dosing regimen used in the Phase III studies), has been reported to be approximately 50–60 nmol/L [8, 41] with a mean plasma AUC_0–24h_ in the range of 6–7 µmol × h/L [41, 42]. Taken together, this suggests that MIV-711 can produce consistent anti-resorptive effects at low circulating plasma levels indicating a sustained level of inhibition of osteoclast-derived cathepsin K. A compound producing a high level of efficacy at low circulating concentrations may be important when targeting cathepsin K. The cathepsin K inhibitor balicatib failed in clinical development due to rash and morphea-like skin reactions [43]. This has been claimed to be due to balicatib’s lysosomotropic properties [44], the rationale being that non-acidic inhibitors like balicatib would accumulate in acidic organelles and lose selectivity towards other cathepsins. However, the cathepsin K inhibitor ONO-5334 is relatively non-selective for cathepsin K when compared to balicatib, odanacatib and MIV-711 [45], but appears to be well-tolerated when given to post-menopausal osteoporotic women for 24 months [46]. This speaks against possible loss of cathepsin K selectivity being associated with side effects. Furthermore, it has been reported that odanacatib, which is acidic and non-lysosomotropic, was associated with increased risk of stroke and morphea-like skin lesions in a large phase III trial [14, 47]. Cathepsin K is present in other tissues like lung and skin. However, life-long deficiency of cathepsin K in pycnodysostosis patients is not associated with any obvious disorders in these organs to our knowledge. It is currently unknown if the side effects of these cathepsin K inhibitors are due to cathepsin K targeting, to their selectivity profile for other cathepsin subtypes, their chemical structure (balicatib and odanacatib have the same electrophilic groups designed to form a covalent bond to the active site cysteine) or to other unknown pharmacological effects. Nevertheless, the diverse nature of the adverse events associated with cathepsin K inhibitors may suggest that these are largely related to their effects on targets other than cathepsins. MIV-711 was well tolerated in the current study and in longer studies in postmenopausal women for up to 28 days, as well as in OA patients for up to 6 months [24, 31]. The most common adverse effects in healthy subjects after dosing with 100 mg MIV-711 once daily for 28 days included skin reactions at ECG electrode sites, headache and gastrointestinal symptoms with comparable incidence after active drug or placebo [31]. In OA patients, the most common adverse effects after treatment with MIV-711 at 100 or 200 mg once daily for 6 months were infrequent musculoskeletal symptoms, infections and rashes [24].

The results demonstrate that MIV-711 is a highly potent, selective, and rapidly reversible inhibitor of human cathepsin K. MIV-711 inhibited the resorptive capacity of human osteoclasts in vitro and also reduced bone resorption in vivo in cynomolgus monkey when given once daily via oral gavage. The anti-resorptive duration in vivo in monkeys outlasted the pharmacokinetics of MIV-711 since a maintained effect (up to 57% reduction) on plasma CTX-I at 24 h post-dose was present despite plasma levels of MIV-711 being at, or below, the level of detection at this time point. MIV-711 also significantly reduced urinary levels of bone resorption biomarkers (CTX-I and NTX-I) as well as CTX-II, a biomarker of cartilage degradation after repeated 5-day dosing. This profile was confirmed in humans where single doses of MIV-711 dose-dependently reduced CTX-I levels by up to 79% at 24 h post dose when concentrations of MIV-711 are minimal. There were no safety or tolerability concerns after single doses and no meaningful effects on the QTcF interval.

## Conclusions

MIV-711 is a potent and selective cathepsin K inhibitor with dose-dependent effects on biomarkers of bone and cartilage degradation in monkey and human. Taken together, MIV-711 shows promise for the treatment of bone and cartilage related disorders in humans such as OA.

## Abbreviations

AMC: 7-Amino-4-methylcoumarin
AUC: Area under the curve
BMD: Bone mineral density
BMI: Body mass index
C_max_: Maximum observed concentration
CTX-I: C-terminal telopeptide I
CTX-II: C-terminal telopeptide II
CYP: Cytochrome P450
DMSO: Dimethyl sulfoxide
DTT: Ditiotreitol
ECG: Electrocardiogram
EDTA: Ethylenediaminetetraacetic acid
EIA: Enzyme immune assay
ELISA: Enzyme-linked immunosorbent assay
IC_50_: Half-maximal inhibitory concentration
K_i_: Inhibitory constant
LC-MS/MS: Liquid chromatography dual mass spectrometry
LLOQ: Lower limit of quantitation
M-CSF: Macrophage colony stimulating factor 1
MRI: Magnetic resonance imaging
NTX-I: N-terminal telopeptide I
OA: Osteoarthritis
p.o.: Per oral
PEG: Polyethylene glycol
PK: Pharmacokinetics
QTcF: QT interval corrected for heart rate according to Fridericia’s formula
RANK: Receptor activator of nuclear factor kappa-B
SEM: Standard error mean
t_1/2_: Half-life
t_max_: Time to maximum observed concentration
WOMAC: Western Ontario and McMaster Universities Osteoarthritis Index

## References

[1] Drake FH, Dodds RA, James IE, Connor JR, Debouck C, Richardson S, Lee-Rykaczewski E, Coleman L, Rieman D, Barthlow R, Hastings G, Gowen M (1996). Cathepsin K, but not cathepsins B, L, or S, is abundantly expressed in human osteoclasts. J Biol Chem.

[2] Väänänen HK, Zhao H, Mulari M, Halleen JM (2000). The cell biology of osteoclast function. J Cell Sci.

[3] Brömme D, Okamoto K, Wang BB, Biroc S (1996). Human cathepsin O_2_, a matrix protein-degrading cysteine protease expressed in osteoclasts. Functional expression of human cathepsin O_2_ in *Spodoptera frugiperda* and characterization of the enzyme. J Biol Chem.

[4] Saftig P, Hunziker E, Wehmeyer O, Jones S, Boyde A, Rommerskirch W, Moritz JD, Schu P, von Figura K (1998). Impaired osteoclastic bone resorption leads to osteopetrosis in cathepsin K-deficient mice. Proc Natl Acad Sci.

[5] Gelb BD, Shi GP, Chapman HA, Desnick RJ (1996). Pycnodysostosis, a lysosomal disease caused by cathepsin K deficiency. Science.

[6] Wijkmans J, Gossen J (2011). Inhibitors of cathepsin K: a patent review (2004–2010). Expert Opin Ther Pat.

[7] Kumar S, Dare L, Vasko-Moser JA, James IE, Blake SM, Rickard DJ, Hwang S-M, Tomaszek T, Yamashita DS, Marquis RW, Oh H, Jeong JU, Veber DF, Gowen M, Lark MW, Stroup G (2007). A highly potent inhibitor of cathepsin K (relacatib) reduces biomarkers of bone resorption both in vitro and in an acute model of elevated bone turnover in vivo in monkeys. Bone.

[8] Stoch SA, Zajic S, Stone J, Miller DL, Van Dyck K, Gutierrez MJ, De Decker M, Liu L, Liu Q, Scott BB, Panebianco D, Jin B, Duong LT, Gottesdiener K, Wagner JA (2009). Effect of the cathepsin K inhibitor odanacatib on bone resorption biomarkers in healthy postmenopausal women: two double-blind, randomized, placebo-controlled phase I studies. Clin Pharmacol Ther.

[9] Nagase S, Ohyama M, Hashimoto Y, Small M, Kuwayama T, Deacon S (2012). Pharmacodynamic effects on biochemical markers of bone turnover and pharmacokinetics of the cathepsin K inhibitor, ONO-5334, in an ascending multiple-dose, phase 1 study. J Clin Pharmacol.

[10] Jerome C, Missbach M, Gamse R (2011). Balicatib, a cathepsin K inhibitor, stimulates periosteal bone formation in monkeys. Osteoporos Int.

[11] Masarachia PJ, Pennypacker BL, Pickarski M, Scott KR, Wesolowski GA, Smith SY, Samadfam R, Goetzmann JE, Scott BB, Kimmel DB, Duong LT (2012). Odanacatib reduces bone turnover and increases bone mass in the lumbar spine of skeletally mature ovariectomized rhesus monkeys. J Bone Miner Res.

[12] Eisman JA, Bone HG, Hosking DJ, McClung MR, Reid IR, Rizzoli R, Resch H, Verbruggen N, Hustad CM, DaSilva C, Petrovic R, Santora AC, Ince BA, Lombardi A (2011). Odanacatib in the treatment of postmenopausal women with low bone mineral density: three-year continued therapy and resolution of effect. J Bone Miner Res.

[13] Eastell R, Nagase S, Ohyama M, Small M, Sawyer J, Boonen S, Spector T, Kuwayama T, Deacon S (2011). Safety and efficacy of the cathepsin K inhibitor ONO-5334 in postmenopausal osteoporosis: the ocean study. J Bone Min Res.

[14] Chapurlat R (2016). Cathepsin K inhibitors and antisclerostin antibodies. The next treatments for osteoporosis?. Joint Bone Spine..

[15] Burr DB, Gallant MA (2012). Bone remodeling in osteoarthritis. Nat Rev Rheumatol.

[16] Karsdal MA, Bay-Jensen AC, Lories RJ, Abramson S, Spector T, Pastoureau P, Christiansen C, Attur M, Henriksen K, Goldring SR, Kraus V (2014). The coupling of bone and cartilage turnover in osteoarthritis: opportunities for bone antiresorptives and anabolics as potential treatments?. Ann Rheum Dis.

[17] Connor JR, LePage C, Swift BA, Yamashita D, Bendele AM, Maul D, Kumar S (2009). Protective effects of a cathepsin K inhibitor, SB-553484, in the canine partial medial meniscectomy model of osteoarthritis. Osteoarthr Cartil.

[18] McDougall JJ, Schuelert N, Bower J (2010). Cathepsin K inhibition reduces CTXII levels and joint pain in the guinea pig model of spontaneous osteoarthritis. Osteoarthr Cartil.

[19] Hayami T, Zhuo Y, Wesolowski GA, Pickarski M, Duong LT (2012). Inhibition of cathepsin K reduces cartilage degeneration in the anterior cruciate ligament transection rabbit and murine models of osteoarthritis. Bone.

[20] Herrero-Beaumont G, Roman-Blas JA (2013). Osteoarthritis: osteoporotic OA: a reasonable target for bone-acting agents. Nat Rev Rheumatol.

[21] Reginster JY (2014). Efficacy and safety of strontium ranelate in the treatment of knee osteoarthritis: results of a double-blind randomized, placebo-controlled trial. Ann Rheum Dis.

[22] Spector TD, Conaghan PG, Buckland-Wright JC, Garnero P, Cline GA, Beary JF, Valent DJ, Meyer JM (2005). Effect of risedronate on joint structure and symptoms of knee osteoarthritis: results of the BRISK randomized, controlled trial. Arthritis Res Ther.

[23] Manicourt DH, Azria M, Mindeholm L, Thonar EJ, Devogelaer JP (2006). Oral salmon calcitonin reduces Lequesne´s algofunctional index scores and decreases urinary and serum levels of biomarkers of joint metabolism in knee osteoarthritis. Arthritis Rheum.

[24] Conaghan PG, Bowes MA, Kingsbury SR, Brett A, Guillard G, Jansson Å, Wadell C, Bethell R, Öhd J. MIV-711, a novel cathepsin K inhibitor demonstrates evidence of osteoarthritis structure modification: results from a 6 month randomized double-blind placebo-controlled Phase IIA trial. American College of Rheumatology annual meeting, 2017; Abstract no. 14L.

[25] Fuller K, Lindstrom E, Edlund M, Henderson I, Grabowska U, Szewczyk KA, Moss R, Samuelsson B, Chambers TJ (2010). The resorptive apparatus of osteoclasts supports lysosomotropism and increases potency of basic versus non-basic inhibitors of cathepsin K. Bone.

[26] Morrison JF, Walsh CT (1988). The behavior and significance of slow binding enzyme inhibitors. Adv Enzymol Relat Areas Mol Biol.

[27] McQueney MS, Field J, Hanning CR, Brun K, Ramachandran K, Connor J, Drake F, Jones CS, Amegadzie BY (1998). Cynomolgus monkey (*Macaca fascicularis*) cathepsin K: cloning, expression, purification and activation. Protein Expr Purif.

[28] Qvist P, Christgau S, Pedersen BJ, Schlemmer A, Christiansen C (2002). Circadian variation in the serum concentration of C-terminal telopeptide of type I collagen (serum CTx): effects of gender, age, menopausal status, posture, daylight, serum cortisol, and fasting. Bone.

[29] Atley LM, Mort JS, Lalumiere M, Eyre DR (2000). Proteolysis of human bone collagen by cathepsin K: characterization of the cleavage site generated by cross-linked N-telopeptide neoepitope. Bone.

[30] Garnero P, Ferreras M, Karsdal MA, Nicamhlaoibh R, Risteli J, Borel O, Qvist P, Delmas PD, Foged NT, Delaisse JM (2003). The type I collagen fragments ICTP and CTX reveal distinct enzymatic pathways of bone collagen degradation. J Bone Miner Res.

[31] Grabowska U, Lindstrom E, Jerling M, Edenius C (2014). MIV-711, a highly selective cathepsin K inhibitor: safety, pharmacokinetics and pharmacodynamics of multiple oral doses in healthy postmenopausal women. Bone Abst.

[32] Dodds RA, Connor JR, Drake FH, Gowen M (1999). Expression of cathepsin K messenger RNA in giant cells and their precursors in human osteoarthritic synovial tissues. Arthritis Rheum.

[33] Hou WS, Li W, Keyszer G, Weber E, Levy R, Klein MJ, Gravallese EM, Goldring SR, Brömme D (2002). Comparison of cathepsins K and S expression within the rheumatoid and osteoarthritic synovium. Arthritis Rheum.

[34] Kafienah W, Brömme D, Buttle DJ, Croucher LJ, Hollander AP (1998). Human cathepsin K cleaves native type I and II collagens at the N-terminal end of the triple helix. Biochem J.

[35] Dejica VM, Mort SM, Laverty S, Percival MD, Antoniou J, Zukor DJ, Poole AR (2008). Cleavage of type II collagen by cathepsin K in human osteoarthritic cartilage. Am J Pathol.

[36] Oestergaard S, Chouinard L, Doyle N, Karsdal MA, Smith SY, Qvist P, Tanko LB (2006). The utility of measuring C-terminal telopeptides of collagen type II (CTX-II) in serum and synovial fluid samples for estimation of articular cartilage status in experimental models of destructive joint diseases. Osteoarthr Cartil.

[37] Grabowska U, Jerling M, Böttiger D, Larsson T, Danielson K, Lindström E, Edenius C (2013). MIV-711, a highly selective cathepsin K inhibitor, first in man study—safety, pharmacokinetics and pharmacodynamics of multiple ascending oral doses in healthy subjects. J Bone Min Res.

[38] Kraus VB, Burnett B, Coindreau J, Cottrell S, Eyre D, Gendreau M, Gardiner J, Garnero P, Hardin J, Henrotin Y, Heinegård D, Ko A, Lohmander LS, Matthews G, Menetski J, Moskowitz R, Persiani S, Poole AR, Rousseau J-C, Todman M (2011). Application of biomarkers in the development of drugs intended for the treatment of osteoarthritis. Osteoarthr Cartil.

[39] Kraus VB, Collins JE, Hargrove D, Losina E, Nevitt M, Katz JN, Wang SX, Sandell LJ, Hoffmann SC, Hunter DJ (2017). Predictive validity of biochemical biomarkers in knee osteoarthritis: data from the FNIH OA biomarkers consortium. Ann Rheum Dis.

[40] Lindström E, Rizoska B, Tunblad K, Edenius C, Bendele AM, Maul D, Larson M, Shah N, Yoder Otto V, Jerome C, Grabowska U (2018). The selective cathepsin K inhibitor MIV-711 attenuates joint pathology in experimental animal models of osteoarthritis.. J Transl Med.

[41] Stoch SA, Zajic S, Stone JA, Miller DL, Bortel L, Lasseter KC, Pramanik B, Cilissen C, Liu Q, Liu L, Scott BB, Panebianco D, Ding Y, Gottesdiener K, Wagner JA (2013). Odanacatib, a selective cathepsin K inhibitor to treat osteoporosis: safety, tolerability, pharmacokinetics and pharmacodynamics—results from single oral dose studies in healthy volunteers. Br J Clin Pharmacol.

[42] Pennypacker BL, Chen CM, Zheng H, Shih M-S, Belfast M, Samadfam R, Duong LT (2014). Inhibition of cathepsin K increases modeling-based bone formation, and improves cortical dimension and strength in adult ovariectomized monkeys. J Bone Miner Res.

[43] Rünger TM, Adami S, Benhamou CL, Czerwinski E, Farrerons J, Kendler DL, Mindeholm L, Realdi G, Roux C, Smith V (2012). Morphea-like skin reactions in patients treated with the cathepsin K inhibitor balicatib. J Am Acad Dermatol.

[44] Falgueyret JP, Desmarais S, Oballa R, Black WC, Cromlish W, Khougaz K, Lamontagne S, Massé F, Riendeau D, Toulmond S, Percival MD (2005). Lysosomotropism of basic cathepsin K inhibitors contributes to increased cellular potencies against off-target cathepsins and reduced functional selectivity. J Med Chem.

[45] Ochi Y, Yamada H, Mori H, Nakanishi Y, Nishikawa S, Kayasuga R, Kawada N, Kunishige A, Hashimoto Y, Tanaka M, Sugitani M, Kawabata K (2011). Effects of ONO-5334, a novel orally-active inhibitor of cathepsin K, on bone metabolism. Bone.

[46] Eastell R, Nagase S, Small M, Boonen S, Spector T, Ohyama M, Kuwayama T, Deacon S (2014). Effect of ONO-5334 on bone mineral density and biochemical markers of bone turnover in postmenopausal osteoporosis: 2-year results from the ocean study. J Bone Miner Res.

[47] Khosla S, Hofbauer LC (2017). Osteoporosis treatment: recent developments and ongoing challenges. Lancet Diabetes Endocrinol.

